# Far-Infrared Therapy Improves Arteriovenous Fistula Patency and Decreases Plasma Asymmetric Dimethylarginine in Patients with Advanced Diabetic Kidney Disease: A Prospective Randomized Controlled Trial

**DOI:** 10.3390/jcm11144168

**Published:** 2022-07-18

**Authors:** Chun-Fan Chen, Chiu-Yang Lee, Fu-An Chen, Chih-Yu Yang, Tz-Heng Chen, Shuo-Ming Ou, Kuo-Hua Lee, Ching-Po Li, Chia-Hao Chan, Pui-Ching Lee, Yung-Tai Chen, Tsung-Lun Lee, Yang Ho, Fan-Yu Chen, Hao-Wei Ma, Jinn-Yang Chen, Ann Charis Tan, Szu-Yuan Li, Chih-Ching Lin

**Affiliations:** 1 School of Medicine, National Yang Ming Chiao Tung University, Hsinchu 300, Taiwan; b8701004@gmail.com (C.-F.C.); fachenymuh@gmail.com (F.-A.C.); s19401021@gmail.com (T.-H.C.); okokyytt@gmail.com (S.-M.O.); s19701093@hotmail.com (C.-P.L.); ytchen0117@gmail.com (Y.-T.C.); leeal274@hotmail.com (T.-L.L.); revasculization@gmail.com (Y.H.); nono007tw@gmail.com (F.-Y.C.); adam055565@hotmail.com (H.-W.M.); jychen@vghtpe.gov.tw (J.-Y.C.); 2 Division of Nephrology, Department of Internal Medicine, National Yang Ming Chiao Tung University Hospital, Yilan 260, Taiwan; 3 Division of Cardiovascular Surgery, Department of Surgery, Taipei Veterans General Hospital, Taipei 112, Taiwan; davidlee0501@gmail.com; 4 Institute of Clinical Medicine, School of Medicine, National Yang Ming Chiao Tung University, Hsinchu 300, Taiwan; cyyang3@vghtpe.gov.tw (C.-Y.Y.); dadabim3520@gmail.com (K.-H.L.); 5 Division of Nephrology, Department of Medicine, Taipei Veterans General Hospital, Taipei 112, Taiwan; box033@gmail.com; 6 Center for Intelligent Drug Systems and Smart Bio-devices (IDS2B), National Yang Ming Chiao Tung University, Hsinchu 300, Taiwan; 7 Stem Cell Research Center, National Yang Ming Chiao Tung University, Hsinchu 300, Taiwan; 8 Division of Nephrology, Department of Medicine, Taipei Veterans General Hospital Yuli Branch, Hualien 981, Taiwan; 9 Department of Medicine, Taipei Veterans General Hospital, Taipei 112, Taiwan; pclee@vghtpe.gov.tw; 10 Division of Nephrology, Department of Internal Medicine, Taipei City Hospital Heping Fuyou Branch, Taipei 100, Taiwan; 11 Center for General Education, National Taipei University, Taipei 104, Taiwan; 12 Department of Nephrology, Fooyin University Hospital, Pingtung 928, Taiwan

**Keywords:** access blood flow, Asymmetric dimethylarginine, arteriovenous fistula, chronic kidney disease, diabetic kidney disease, far-infrared therapy

## Abstract

Asymmetric dimethylarginine (ADMA) is an endogenous inhibitor of nitric oxide synthase and plays a significant role in the pathogenesis of arteriovenous fistula (AVF) dysfunction. The aim of this study is to evaluate the effect of far-infrared (FIR) therapy on the maturation and patency of newly-created AVFs in patients with advanced diabetic kidney disease (DKD) as well as the concurrent change in plasma ADMA. The study enrolled 144 participants with advanced DKD where 101 patients were randomly allocated to the FIR therapy group (N = 50) and control group (N = 51). Patients receiving FIR therapy had a decreased AVF failure rate within 12 months (16% versus 35.3%; *p* = 0.027); decreased incremental change of ADMA concentration at the 3rd and 12th month; increased AVF blood flow at the 1st, 3rd, and 12th month; increased 3-month physiologic maturation rate (88% versus 68.6%; *p* = 0.034); increased 1-year unassisted AVF patency rate (84% versus 64.7%; *p* = 0.017); and increased clinical AVF maturation rate within 12 months (84% versus 62.7%; *p* = 0.029) compared to the control group. The study demonstrates that FIR therapy can reduce the incremental changes in plasma ADMA concentration, which may be associated with the improvement of AVF prognosis in patients with advanced DKD.

## 1. Introduction

Asymmetric dimethylarginine (ADMA) has been characterized as an endogenous inhibitor of nitric oxide synthase by competing with l-arginine, leading to vasoconstriction and endothelial dysfunction [1,2]. In comparison with normal individuals, ADMA plasma concentration may be 2- to 6-times higher in patients with chronic kidney disease (CKD) and end-stage renal disease (ESRD), and ADMA is recognized as one of the most crucial uremic toxins [3,4]. ADMA is demonstrated to be a significant risk factor for atherosclerosis, cardiovascular outcomes, and all-cause mortality among both normal individuals and CKD patients [1,5,6]. Higher plasma ADMA concentrations may also strongly predict the risk of restenosis for stenotic arteriovenous fistula (AVF) in hemodialysis (HD) patients after undergoing angioplasty [7]. However, the role of ADMA in the maturation of newly-created AVFs in ESRD patients remains unknown.

Creating and maintaining well-functioning vascular access has been one of the main goals for high-quality dialysis, and AVF has been the preferred HD access with a higher patency rate. Unfortunately, poor AVF maturation compels some patients to prolong the time of dependence on the central venous catheter and contributes to risks from catheter use. Balloon-assisted angioplasty is utilized to improve the function of failing to mature AVF, but this procedure may potentially result in endothelial injury [8]. Far-infrared (FIR) therapy is an alternative non-invasive treatment that is confirmed to increase blood flow, as well as enhance the maturation of newly-created AVFs [9]. FIR is an invisible electromagnetic wave with a wide wavelength range between 3 to 100 μm [10]. AVF blood flow and patency have improved when treated with FIR not only via thermal effect-induced regional vasodilation but also through non-thermal effects in improving endothelial function [11,12,13,14]. The beneficial effects of FIR therapy in promoting AVF maturation may be associated with either regional or systemic ADMA concentrations. We hypothesize that plasma ADMA concentrations could be related to the future maturation of newly-created AVFs, and the influence may be regulated by FIR therapy. We aim to investigate the effect of FIR on the maturation and patency of newly-created AVFs in patients with advanced DKD and the concurrent change in plasma ADMA concentrations.

## 2. Materials and Methods

### 2.1. Study Design and Patient Selection

The study is a randomized controlled trial where the study sample consisted of patients aged between 18 and 80 years old who met the following inclusion criteria: (1) clinically diagnosed with advanced diabetic kidney disease (DKD), which is defined by having diabetic retinopathy and a history of diabetes mellitus for more than 5 years; (2) estimated glomerular filtration rate between 5 and 20 mL/min/1.73m^2^ body surface area; (3) plan to receive pre-emptive AVF creation of venous-end-to-arterial-side anastomosis in the upper extremity; and (4) not expected to receive either dialysis or renal transplantation within the following 3 months after participating in the study. These participants were receiving regular medical follow-ups in the nephrology outpatient clinic of Taipei Veterans General Hospital and were enrolled in the CKD care program. Patients who met the following conditions were excluded from the study, including: (1) creation of arteriovenous graft or tunneled cuffed HD catheter as permanent vascular access type; (2) unwilling to undergo HD; (3) heart failure with New York Heart Association class III or IV; (4) episode of cardio- or cerebrovascular event or receiving intervention therapy within 3 months before screening; and (5) terminal cancer with life expectancy less than 1 year. All patients gave their informed consent for inclusion before they participated in the study. The study was conducted in accordance with the Declaration of Helsinki, and the protocol was approved by the Institutional Review Board of Taipei Veterans General Hospital (ClinicalTrials.gov identifier: NCT01138254; date of registration: 7 June 2010). Patients were recruited from November 2008 to August 2010. Follow-up on patients continued until a year after in August 2011. The patients were asked to maintain their regular medications and were taught the standardized upper extremity exercise for AVF maturation. The baseline characteristics of patients were directly obtained from the patient and patient records from the CKD care program. After enrollment, through simple randomization using computer-generated numbers, the patients were classified into two groups with the allocation ratio of 1:1, namely the FIR group, where patients received FIR therapy for 12 months, and the control group, where they only underwent upper extremity exercise for AVF maturation. 

### 2.2. Study Protocol

The patients in the experimental group underwent FIR therapy for 40 min per session, 3 times per week, starting from the second day after AVF creation and continued for 12 months. The top radiator was set at a height of approximately 25 cm above the surface of the AVF. A WSTM TY101 FIR emitter (WS Far IR Medical Technology Co., Ltd., Taipei, Taiwan) was used for the treatment, with the wavelength of the machine ranging between 3 and 25 μm (a peak at 5 to 6 μm). These patients received FIR therapy either at home or the nephrology outpatient clinic before transitioning to the ESRD phase and starting HD (using high-efficiency and high-flux dialyzers), after which they then received FIR therapy for 40 min 3 times per week during HD. 

All patients had their access blood flow (Qa) measured by the same ultrasound surveyor via color Doppler ultrasonography at 4 time points, namely during the 2nd day, 1st month, 3rd month, and 12th month after AVF creation and labeled as Qa0, Qa1, Qa3, and Qa12, respectively. The velocity and Qa were determined by the spectral analysis of flow at the site approximately 3 cm upstream from the AVF anastomotic site. The ultrasonic surveyor was blinded to the status of the assignment for each participant. Color Doppler ultrasonography (Model SSA 340A; Toshiba, Tokyo, Japan) with a 7.5-MHz linear array transducer was used for the Qa measurements. Asymmetric dimethylarginine, a marker of endothelial function, was measured at 3 time points, namely immediately before AVF creation, as well as 3 months and 12 months after AVF creation, respectively. Plasma ADMA concentrations were determined with commercially available enzyme-linked immunosorbent assay kits (DLD Diagnostika, Hamburg, Germany) [15]. The correlation coefficient between liquid chromatography–mass spectrometry ADMA and enzyme-linked immunosorbent assay ADMA is 0.98. The recovery rate for ADMA was 90%, and the between-assay and within-assay variation coefficients were less than 8% and 7%, respectively. Blood samples were collected from patients before AVF creation to measure the biochemical parameters and were determined by an autoanalyzer (Model 7600-310; Hitachi, Tokyo, Japan). The estimated glomerular filtration rate was calculated according to the simplified version of the equation used in the Modification of Diet in Renal Disease study, which was further modified by Ma et al. for Chinese patients with CKD (estimated glomerular filtration rate = 175 × plasma creatinine − 1.234 × age − 0.179 × 0.79 [if female] [16].

The primary outcome is the rate of AVF failures occurring within 12 months after creation and the concurrent change in plasma ADMA concentration and AVF failure is defined as having thrombotic AVF with insufficient blood flow in patients not undergoing HD, or requiring any intervention (angioplasty or surgery) to maintain AVF patency in patients undergoing HD. The secondary outcomes included: (1) primary unassisted AVF patency defined as the percentage free from AVF dysfunction (e.g., thrombosis or special circumstances requiring interventional procedures) with the censoring criteria of death with functioning AVF, undergoing renal transplantation, or shifting to peritoneal dialysis; (2) AVF physiological maturation defined as AVF Qa ≥500 mL/min at 3 months after creation without the assistance of any interventional procedures; and (3) AVF clinical maturation within 1 year, defined as the ability to place 2 needles on the AVF for HD with an extracorporeal blood flow rate ≥ 200 mL/min during 8 of the 12 HD sessions during a 30-day suitability ascertainment period. The patient’s physician makes the decision when to start HD or when there is the need for AVF interventional therapy. 

In the 2007 study on patients receiving maintenance HD, there were 24 patients with underlying diabetes mellitus in the control group, 10 of which experienced AVF failure (41.7%), while there are 25 patients with underlying diabetes mellitus in the FIR group, 4 of which experienced AVF failure (16%) [17]. In the 2013 study on CKD patients, 14.3% (4 out of 28) of the diabetic patients in the FIR group and 43.5% (10 out of 23) of the diabetic patients in the control group experienced AVF failure [9]. A 2018 meta-analysis of 23 studies showed that a statistically significant higher rate of AVF failure was found in diabetic patients compared with non-diabetic patients (odds ratio = 1.682; 95% confidence interval = 1.429–1.981, *p* < 0.001) [18]. Combining the results from the aforementioned previous studies, we have set the rate of newly-created AVF failures of DKD patients occurring within 12 months as 15% (close to the mean of 16% and 14.3%) in the FIR group and 42% (close to the mean of 41.7 and 43.5%) in the control group, respectively [17].

The sample size estimation sets type I error as 0.05 and type II error as 0.20 to detect the rate of newly-created AVF failures of DKD patients occurring within 12 months as 15% and 42% in the FIR group and control group, respectively. The rates are based on the assumption that the malfunction rate of newly-created AVFs in the FIR group and control group is 12% and 30%, respectively. DKD accounted for 40% of ESRD cause in the general population, a higher rate of AVF failure in diabetic patients compared with non-diabetic patients, and a dropout rate of 10% [9]. Thus, the number of patients needed to complete the study should be more than 50 in each group.

### 2.3. Statistical Analysis

Statistical analysis was performed using the SPSS statistical software for Windows (Version 21.0; IBM Corp., Armonk, NY, USA). The distributions of continuous variables in groups were expressed as the mean ± standard deviation and compared using Student’s *t*-test. All data were first tested to determine whether they are normally distributed before being analyzed. Categorical variables were expressed as percentages and analyzed using a chi-square test. The Kaplan–Meier estimate was used to measure the unassisted AVF patency rate at 12 months, and the log-rank test was used to examine the differences between these 2 treatment groups. A significant value of *p* < 0.05 was set.

## 3. Results

### 3.1. Patient Characteristics

**Figure 1:**
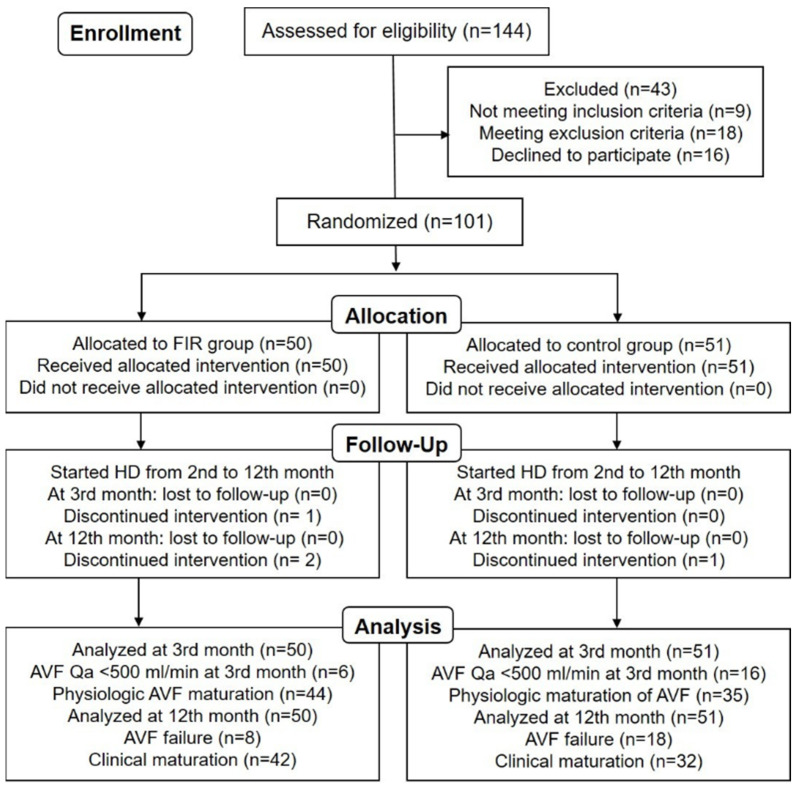
Study enrollment flow chart of participants in the randomized controlled trial to evaluate the effect of FIR therapy on AVF Qa and plasma ADMA concentrations in advanced DKD patients. Abbreviations: FIR, far-infrared; HD, hemodialysis; AVF, arteriovenous fistula; Qa, access blood flow; ADMA, asymmetric dimethylarginine; DKD, diabetic kidney disease.

The study initially enrolled 144 patients, and 43 patients were excluded due to not meeting the inclusion criteria (n = 9), meeting the exclusion criteria (n = 18), and declining to participate in the study (n = 16). In the final analysis, there were 101 patients randomly allocated to two groups, namely the experimental (FIR) group (n = 50) and the control group (n = 51) (Figure 1). There was no significant intergroup difference in the baseline clinical characteristics (Table 1). The patients started to undergo HD during the first year, starting from the 2nd month onwards. During the study period, there were 1 and 2 patients who discontinued FIR therapy on the 3rd and 12th month, respectively. One patient each died in the FIR and control groups and no patients were lost to follow-up. There were 51 patients in the control group and 50 patients in the FIR group during the 12-month clinical trial. 

### 3.2. The Effect of 12-Month FIR Therapy on Qa and Plasma ADMA Concentrations in HD Patients

**Figure 2:**
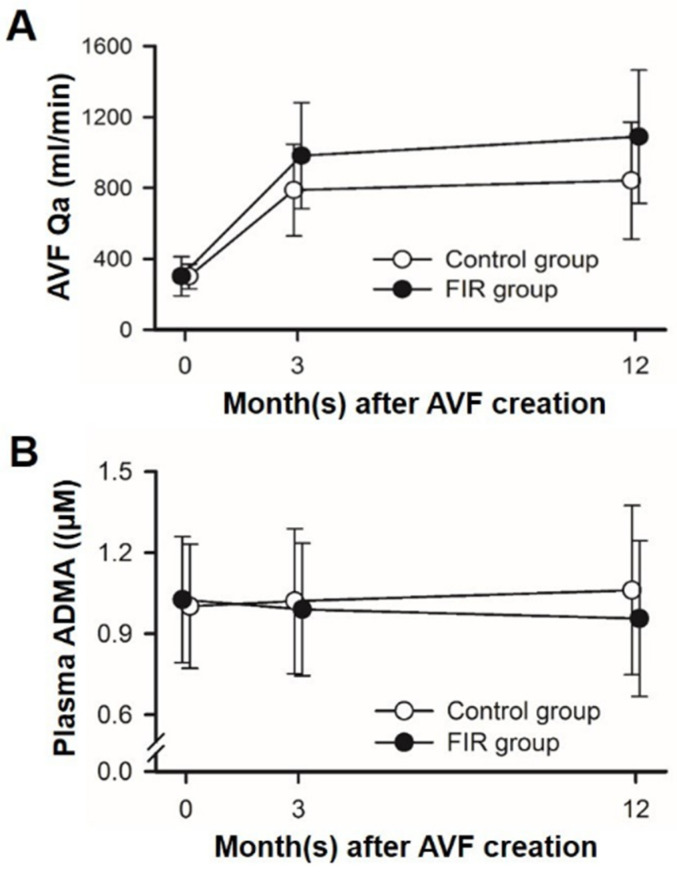
(**A**) AVF Qa on the 2nd day (labeled as 0), 3rd month, and 12th month after AVF creation; (**B**) Plasma ADMA concentrations on the 2nd day (labeled as 0), 3rd month, and 12th month after AVF creation. In comparison with the control group (white circles), AVF Qa in FIR group (black circles) are similarly distributed at most given concentrations of plasma ADMA before FIR therapy, but are relatively higher on the 3rd month as well as on the 12th month. Abbreviations: ADMA, asymmetric dimethylarginine; AVF, arteriovenous fistula; Qa, access blood flow; FIR, far-infrared.

After AVF creation and before the start of FIR therapy, there was no significant difference in the post-operative Qa on the second day between the treatment and control groups (Qa_0_: 302.2 ± 110.1 vs. 301.0 ± 70.0 mL/min, respectively; *p* = 0.947). By the end of the 12-month study period, patients in the FIR group had significantly higher Qa measurements in comparison to the patients in the control group, with the results of (1) Qa_1_: (761.8 ± 276.9 vs. 596.5 ± 216 mL/min, respectively; *p* = 0.001), (2) Qa_3_: (982.2 ± 299.4 vs. 787.5 ± 259.1 mL/min, respectively; *p* = 0.001), and (3) Qa_12_: (1089.4 ± 376.2 vs. 842.0 ± 330.1 mL/min, respectively; *p* = 0.001) (Figure 2A). Regarding the effect of FIR therapy on ADMA concentrations, the incremental change of plasma ADMA concentrations in the FIR group was significantly lower than that in the control group from the pre-operative period to the 3rd month (−0.036 ± 0.047 vs. 0.019 ± 0.050 mL/min, respectively; *p* = 0.01), as well as from the pre-operative period to the 12th month (−0.070 ± 0.087 vs. 0.060 ± 0.096 mL/min, respectively; *p* = 0.001), even though there was no difference in the plasma ADMA concentrations at these 3 different time points (Table 2 and Figure 2B).

### 3.3. The Effect of FIR Therapy on AVF Maturation and Patency

**Figure 3:**
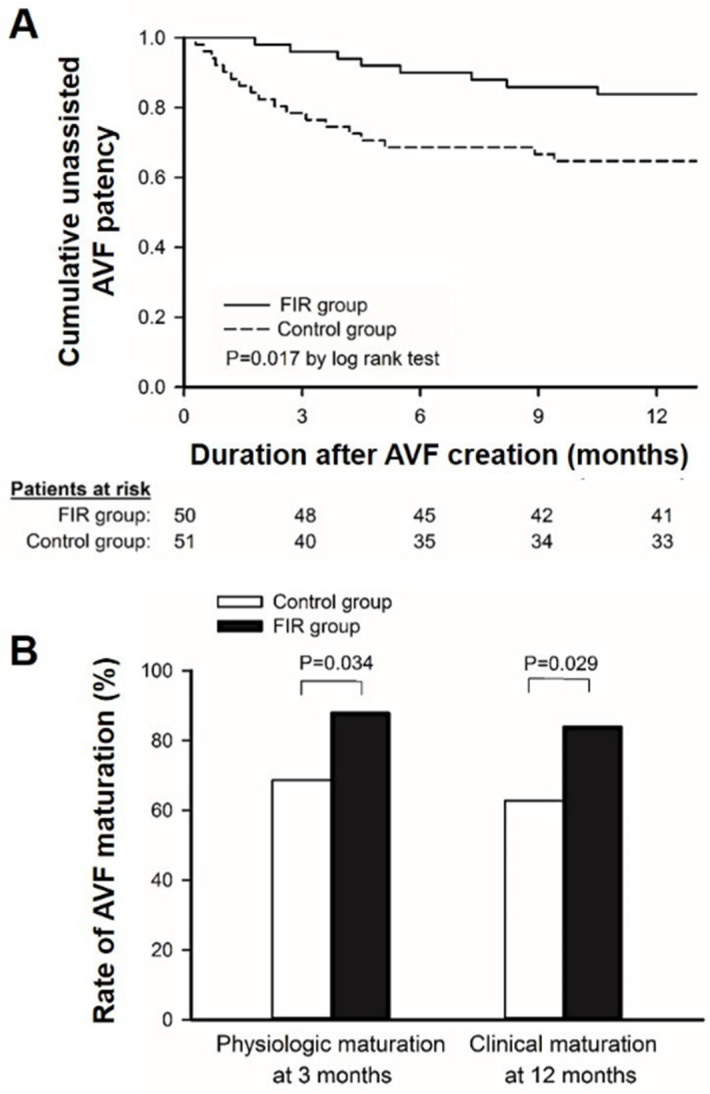
(**A**). Survival curves for cumulative unassisted AVF patency on the 12th month; (**B**) comparison of physiologic AVF maturation rates on the 3rd month and clinical AVF maturation on the 12th month. Abbreviations: FIR, far-infrared; AVF, arteriovenous fistula; DKD, diabetic kidney disease.

In Table 2, patients in the FIR group experienced significantly lower rates of AVF failure in comparison with the control group (16% vs. 35.3%, respectively; *p* = 0.027). More patients in the control group experienced AVF occlusion compared to the FIR group before the first HD session (13.7% vs. 2%, respectively; *p* = 0.029), while the need for intervention of patients already undergoing HD showed an insignificant difference (21.6% vs. 14%, respectively; *p* = 0.32). The patients in the experimental group showed improved secondary outcomes after FIR therapy for 12 months, including a higher cumulative unassisted patency rate at 12 months (84% vs. 64.7%, respectively; *p* = 0.017) (Figure 3A), unassisted physiologic maturation on the 3rd month (88.0% vs. 68.6%, respectively; *p* = 0.034), and clinical maturation where AVF is well-functioning for HD at 12 months (84% vs. 62.7%, respectively; *p* = 0.029) (Figure 3B). No patients in the experimental group complained of any adverse effects associated with FIR therapy.

### 3.4. AVF Prognosis and the Change in Plasma ADMA over Time

Since ADMA is integral to AVF patency and maturation, we further subdivided the patients into three subgroups according to their AVF prognosis (occlusion group, intervention group, and stable group) and compared their plasma ADMA concentrations over three time points during the study period (Table 3). There were statistically significant differences between the plasma ADMA concentrations at all time points among the subgroups. From enrollment to the end of the study at 12 months, the occlusion group had the highest plasma ADMA concentration, followed by the intervention group, and then the stable group. In addition, the highest patient percentage who underwent FIR therapy among the AVF prognosis subgroups were from the stable group (56%), followed by 38.9% from the intervention group, and 12.5% from the occlusion group.

## 4. Discussion

DKD is the most common cause of ESRD globally, and many patients with DKD have relatively poor vascular conditions, which is not conducive to AVF maturation despite a detailed pre-operative evaluation [18]. FIR therapy was reported to be effective in improving the maturation, access flow, and patency of newly-created AVFs in patients with CKD stages 4 and 5 [9]. In this study, we focused on patients with advanced DKD to evaluate the effect of FIR therapy on DKD patients who have been associated with poor AVF maturation rates. We demonstrated that FIR therapy may improve AVF function by increasing Qa measurements accompanied by a significant reduction in the incremental change of plasma ADMA concentrations. Higher plasma ADMA concentrations were found in patients with poorer AVF prognosis. FIR therapy also significantly decreased the AVF failure rate and improved the unassisted AVF patency rate, as well as the physiological and functional AVF maturity in patients with advanced DKD who account for about 40% of incident ESRD patients in Taiwan. No patients reported thermal injuries related to FIR therapy.

Endogenous ADMA originated from the monomethylation of protein-bound l-arginine by type-I protein arginine methyltransferases and hydrolysis of ubiquitous proteins containing methylated arginine residues [19]. Most of ADMA is hydrolyzed to l-citrulline and dimethylamine by the dimethylarginine dimethylaminohydrolase (DDAH) enzyme, which is abundant in the brain, heart, and kidneys. However, in contrast, kidneys are only responsible for less than 20% of ADMA excretion through the urine [20]. DDAH plays a major role in ADMA metabolism and decreased DDAH activity results in a corresponding increase in endothelial ADMA accumulation, which leads to the inhibition of NO synthesis by competing with l-arginine. In addition to elevated ADMA concentrations in patients with CKD and ESRD, plasma ADMA concentrations are also elevated in patients with type-2 diabetes mellitus, and it has been demonstrated that glucose-induced DDAH impairment causes ADMA accumulation, contributing to endothelial vasodilator dysfunction in diabetes mellitus [21]. Multifactorial factors include reducing the content of DDAH in the kidneys, reducing ADMA excretion in the urine, and reducing DDAH activity in diabetes mellitus, which may lead to increased plasma ADMA concentrations in patients with advanced DKD and impaired endothelial nitric oxide synthase function, thereby making AVF more prone to poor maturation and dysfunction. 

In this study, FIR therapy demonstrated beneficial effects on the maturation and patency of newly-created AVFs. FIR therapy may exert a direct thermal effect to induce vasodilation and increase access blood flow, and the increased access blood flow may in turn increase wall shear stress, stimulate endothelial cells and trigger endothelial nitric oxide synthase activity, and promote AVF vasodilation [22]. Furthermore, FIR therapy may also exert non-thermal effects, including the inhibition of intimal hyperplasia, decrease in oxidative stress, suppression of inflammation, and improvement of endothelial function, to promote AVF maturation [11,12,13,14]. The reason that FIR therapy improves endothelial function may be through decreasing plasma ADMA concentrations. The shear stress in AVF should be higher than that in other vasculatures, which may result in the positive regulation of type-I protein arginine methyltransferases through the NFκB pathway and increased ADMA synthesis and accumulation at a higher rate in AVF than that of other vasculatures in ESRD patients [23]. FIR therapy can induce heme oxygenase-1 mRNA expression via the activation of the NF-E2-related factor-2/antioxidant responsive element complex [13,24]. The increased antioxidant and heme oxygenase-1 expression efficiently neutralized the reactive oxygen species and detoxified toxic chemicals and reduced reactive oxygen species-mediated activation of the NFκB pathway, thereby attenuating the type-I protein arginine methyltransferases activation from increasing shear stress [25]. FIR therapy may exert a potent anti-inflammatory effect on the vascular endothelium through inhibiting the TNF-α-mediated expression of E-selectin, vascular cell adhesion molecule-1, intercellular adhesion molecule-1, monocyte chemoattractant protein-1, and interleukin-8. The inhibition of TNF-α may also reduce the inhibition of DDAH and in turn increase ADMA metabolism, leading to a lower ADMA accumulation in the AVF of the FIR group compared to the control group [13,26]. It is reasonable for long-term FIR therapy to reduce the incremental change of plasma ADMA concentrations in the FIR group compared to the control group. Elevated plasma ADMA concentrations in ESRD patients were associated with endothelial dysfunction, which can be attributed to the following effects from the deprivation of NO production, including leukocyte adhesion, platelet aggregation and adhesion, extracellular matrix formation, and smooth muscle proliferation [19,27]. ADMA has also been reported to inhibit the differentiation, function, and mobilization of endothelial progenitor cells, which may explain in part the pathogenetic mechanism of AVF maturation failure in ESRD patients [28]. Concerning the role of ADMA on AVF patency in HD patients, patients with higher ADMA concentrations had a nearly three-fold higher risk of target lesion restenosis than those with lower concentrations [7]. Even though the plasma ADMA concentrations in the patients in our study were lower than what is qualified to manifest clinical effects, the ADMA concentrations within endothelial cells may be high enough to cause an AVF to fail to mature [26]. 

There are some limitations in our study. First, only the plasma ADMA concentrations of the systemic circulation were examined. Thus, the decrease in plasma ADMA concentration cannot accurately represent the change in ADMA concentration that immediately left the AVF after FIR therapy. However, there is no difference in the basic characteristics between the two groups in this study, and the only intervention between groups is FIR therapy. The significant intergroup difference in plasma ADMA concentrations indirectly demonstrated the effect of FIR therapy on lowering plasma ADMA concentrations. Second, the study only recruited patients with newly-created AVFs but did not include patients who had received newly-created arteriovenous grafts or patients who were already in the uremia stage undergoing HD via AVF. Previous studies had demonstrated the beneficial effects of FIR therapy in AVF patency, as well as the post-angioplasty unassisted patency of arteriovenous grafts in HD patients, but further studies are needed to confirm the association found between these beneficial effects and plasma ADMA concentrations [17,29]. Third, the plasma ADMA concentration was not measured immediately before and after AVF interventions and therefore could not be used to conduct a comparison of the plasma ADMA concentrations of patients who require AVF interventions and those who did not. Fourth, there is no sham treatment for the control group in the study protocol, which can be considered in future studies to reduce unwanted bias. Fifth, the study was conducted in a single center in Taiwan, and the effect of FIR therapy on plasma ADMA concentrations may not be the same with a study sample of other ethnicities due to the variability in associated regulator genes. It is necessary to conduct a large multicenter international trial to confirm the effect of FIR therapy on newly-created AVFs and the corresponding changes in plasma ADMA concentrations. 

In conclusion, FIR therapy is an optimal non-invasive strategy to improve the maturation and patency of newly-created AVFs in patients with advanced DKD, which may partly result from the decrease in the incremental change of plasma ADMA concentrations after FIR therapy.

## Figures and Tables

**Table 1:** Comparison of baseline clinical characteristics between advanced DKD patients with and without FIR therapy for 12 months.

Characteristics	Control Group (n = 51)	FIR Group (n = 50)	*p*
Age (years)	62.3 ± 14.2	62.4 ± 18.9	0.980
Females	45.1	50	0.622
Comorbidities			
Hypertension	66.7	68	0.886
Coronary artery disease	17.6	18	0.963
Peripheral artery disease	5.9	8	0.678
Cerebrovascular disease	3.9	6	0.630
Biochemistry			
Hematocrit (%)	30.9 ± 4.7	30.5 ± 4.8	0.782
Serum creatinine (mg/dL)	7.42 ± 4.41	7.35 ± 3.51	0.927
eGFR (ml/min/1.73 m^2^)	9.02 ± 5.06	9.03 ± 5.69	0.997
Serum calcium (mg/dL)	8.3 ± 1.1	8.4 ± 1.2	0.517
Serum phosphate (mg/dL)	5.3 ± 1.8	5.1 ± 1.6	0.374
Parathyroid hormone (pg/mL)	252.2 ± 211.7	279.7 ± 247.2	0.587
Urine protein creatinine ratio	5.7 ± 3.1	5.9 ± 3.3	0.526
Medications			
Calcium channel blocker	51	40	0.268
Angiotensin II receptor blocker	47.1	46	0.915
HMG-CoA reductase inhibitor	35.3	34	0.891
Antiplatelet agents	66.7	56	0.271
Pentoxifylline	62.7	54	0.373
Aspirin	21.6	20	0.846
Clopidogrel	5.9	6	0.980

Continuous variables were presented as mean ± standard deviation. Categorical variables were presented as percentages. Abbreviations: DKD, diabetic kidney disease, eGFR, estimated glomerular filtration rate; HMG-CoA reductase inhibitor, 3-hdroxy-3-methyl-glutaryl coenzyme A reductase inhibitor.

**Table 2:** Comparison of plasma ADMA concentrations and Qa, AVF maturation, and patency in advanced DKD patients with and without FIR therapy for 12 months.

Characteristics	Control Group (n = 51)	FIR Group (n = 50)	*p*
Pre-operative diameter of vein for AVF (mm)	3.64 ± 0.96	3.56 ± 0.92	0.756
Radiocephalic AVF	74.5	78	0.680
Renal function and clearance			
eGFR_0_ (mL/min/1.73 m^2^)	9.02 ± 5.06	9.03 ± 5.69	0.997
eGFR_3_ (mL/min/1.73 m^2^)	7.51 ± 3.82	7.69 ± 3.91	0.825
eGFR_12_ (mL/min/1.73 m^2^)	3.54 ± 1.79	3.85 ± 1.96	0.676
Kt/V_12_	1.52 ± 0.15	1.63 ± 0.16	0.513
Plasma ADMA concentrations			
ADMA_0_ (µM)	1.001 ± 0.230	1.026 ± 0.233	0.597
ADMA_3_ (µM)	1.021 ± 0.268	0.990 ± 0.245	0.549
∆ADMA_3-0_ (µM)	0.019 ± 0.050	−0.036 ± 0.047	0.01
ADMA_12_ (µM)	1.061 ± 0.313	0.956 ± 0.288	0.082
∆ADMA_12-0_ (µM)	0.060 ± 0.096	−0.070 ± 0.087	0.001
Qa rates			
Qa_0_ (mL/min)	301.0 ± 70.0	302.2 ± 110.1	0.947
Qa_1_ (mL/min)	596.5 ± 216.0	761.8 ± 276.9	0.001
Qa_3_ (mL/min)	787.5 ± 259.1	982.2 ± 299.4	0.001
Qa_12_ (mL/min)	842.0 ± 330.1	1089.4 ± 376.2	0.006
Primary outcomes			
AVF failure within 12 months	35.3	16	0.027
AVF occlusion on 12th month	13.7	2	0.029
Intervention for AVF within 12 months	21.6	14	0.32
Secondary outcomes			
Unassisted AVF patency on 12th month	64.7	84	0.017
Physiologic AVF maturation on 3rd month	68.6	88	0.034
Clinical AVF maturation within 12 months	62.7	84	0.029

Continuous variables were presented as mean ± standard deviation. Categorical variables were presented as percentages. ADMA_0/3/12_ indicates the measurement of ADMA concentrations one day before, on the 3rd month, and 12th month after AVF creation. Abbreviations: FIR, far-infrared, AVF, arteriovenous fistula, eGFR, estimated glomerular filtration rate, ADMA, asymmetric dimethylarginine, Qa_0_/Qa_1_/Qa_3_/Qa_12_, Qa (access blood flow) measured on the 2nd day, 1st, 3rd, or 12th month after AVF creation.

**Table 3:** Comparison of plasma ADMA concentrations in advanced DKD patients with different AVF prognosis for 12 months.

Plasma ADMA Concentration	AVF Prognosis			*p*
	Occlusion Group (n = 8)	Intervention Group (n = 18)	Stable Group (n = 75)	
ADMA_0_ (µM)	1.142 ± 0.318	1.078 ± 0.299	0.984 ± 0.274	0.042
ADMA_3_ (µM)	1.193 ± 0.352	1.104 ± 0.306	0.962 ± 0.265	0.026
ADMA_12_ (µM)	1.261 ± 0.373	1.146 ± 0.332	0.949 ± 0.247	0.011

Continuous variables were presented as mean ± standard deviation. ADMA_0/3/12_ indicates the measurement of ADMA concentrations one day before, on the 3rd month, and 12th month after AVF creation. Abbreviations: ADMA, asymmetric dimethylarginine, AVF, arteriovenous fistula, FIR, far-infrared.

## Data Availability

Data sharing not applicable.
